# Interprofessional education through shadowing experiences in multi-disciplinary clinical settings

**DOI:** 10.1186/1746-1340-18-31

**Published:** 2010-12-02

**Authors:** John J Riva, Jessica MS Lam, Elizabeth C Stanford, Ainsley E Moore, Andrea R Endicott, Iris E Krawchenko

**Affiliations:** 1 Department of Family Medicine, McMaster University, McMaster Innovation Park, 175 Longwood Rd S, Hamilton, ON, Canada; 2 Toronto Western Hospital, 399 Bathhurst St, Toronto, ON, Canada; 3 Michael G. DeGroote School of Medicine, McMaster University, Niagara Regional Campus, 142 Queenston St, St Catharines, ON, Canada; 4 Ontario Chiropractic Association, 20 Victoria St, Toronto, ON, Canada; 5 Dell Pharmacy, Rosedale Medical Group, 1955 King St E, Hamilton, ON, Canada

## Abstract

The World Health Organization has recently added Interprofessional Education (IPE) to its global health agenda recognizing it as a necessary component of all health professionals' education. We suggest mandatory interprofessional shadowing experiences as a mechanism to be used by chiropractic institutions to address this agenda. IPE initiatives of other professions (pharmacy and medicine) are described along with chiropractic. This relative comparison of professions local to our jurisdiction in Ontario, Canada is made so that the chiropractic profession may take note that they are behind other health care providers in implementing IPE.

Interprofessional shadowing experiences would likely take place in a multi-disciplinary clinical setting. We offer an example of how two separate professions within a Family Health Team (FHT) can work together in such a setting to enhance both student learning and patient care. For adult learners, using interprofessional shadowing experiences with learner-derived and active objectives across diverse health professional groups may help to improve the educational experience. Mandatory interprofessional shadowing experiences for chiropractors during their training can enhance future collaborative practice and provide success in reaching a goal common to each profession - improved patient care.

## Introduction

Interprofessional education (IPE) has been proposed as an important foundation in preparing health professionals for patient care within collaborative care environments. However, mandatory IPE experiences are not the norm in many schools of pharmacy and chiropractic [1,2]. The following definition provided by the Centre for the Advancement of Interprofessional Education (2002) is used to define IPE: "Interprofessional Education occurs when two or more professions learn with, from and about each other to improve collaboration and the quality of care" [3].

Two important goals for collaborative health care models include: optimizing access to the skills and competencies of a wide range of health professionals; and facilitating health promotion and the prevention of illness [4]. Collaborative health care is promoted as a solution to health workforce resource shortages, and as a way of increasing access to and improving the quality of care. As a result, health professional faculties across North America have been focusing their attention on frameworks to integrate IPE programs into their curricula [5,6]. Worldwide, the World Health Organization has added IPE to its global health agenda recognizing it as a necessary component of all health professionals' education [7].

Systematic reviews have been conducted on the introduction and subsequent impact of early interprofessional collaboration on attitudes, perceptions, and willingness of health care providers to engage in cooperative effort for patient care [8]. Although difficult to objectively evaluate IPE through randomized controlled trials, studies have found that a combination of didactic and clinical experiences may positively influence students' attitudes towards interprofessional care [9,10].

An Ontario scoping review in 2009 on IPE found 90 published works that described learning opportunities for medical students, 41 for pharmacy students, and none for chiropractic students. It went on to describe, "It is possible that IPE with chiropractic students is occurring, but is not yet documented in the literature. To be sure, IPE opportunities for chiropractic students in Ontario are limited since students are part of a private institution that only offers chiropractic education" [11].

The teamwork in the domain of health care necessitates a comprehension of the diverse roles of each team member. Mandatory interprofessional shadowing experiences at chiropractic institutions could provide students of chiropractic with opportunities to have interprofessional mentoring, to learn from faculty from diverse disciplines and to interact with groups of interprofessional students and practitioners in clinical settings. These experiences allow for the sharing of knowledge and would stimulate an interprofessional approach that is centered on the patient.

IPE initiatives used by other professions (pharmacy and medicine) are described along with chiropractic in a local jurisdiction of Ontario, Canada for a relative comparison. This is done to highlight to the chiropractic profession that they are behind other health care professions in implementing IPE.

Interprofessional shadowing experiences would most likely take place in a multi-disciplinary clinical setting. We offer an example of how two separate professions within a Family Health Team (FHT) can work together in such a setting to enhance both student learning and patient care. A FHT is a health care organization that includes a team of family physicians, nurse practitioners, registered nurses, social workers, dietitians, and other health care professionals who work together to provide health care for their community [12].

For interprofessional shadowing experiences it is recommended that the educational institution not define the specific site visit objectives. An adult learning approach is favoured. This approach demonstrates the value of letting the learner take an active role in defining the objectives specific to his or her needs. However, the main learning outcomes for this intervention could be explicitly described by the institution to ensure learner participation in these goals, such as identifying profession-specific terminology, benefits and challenges of team-building and interprofessional communication.

## IPE Initiatives at Ontario Schools of Pharmacy and Chiropractic

The University of Toronto established its Centre for IPE in 2006 and has since successfully incorporated a longitudinal curriculum that connects students of the Faculties of Nursing, Medicine, Social Work, Dentistry, Physical Education, and Pharmacy. An evolving framework highlighting the interdependency of IPE and collaborative patient-centered practice illustrates the mandate of IPE [13].

With respect to pain management, the Leslie Dan Faculty of Pharmacy at the University of Toronto has involved its students in IPE events, most notably the annual Interfaculty Pain Curriculum. These events provide students with an opportunity to appreciate the effectiveness of team-based collaboration on patient care and to identify common barriers that may arise in the process.

Regarding chiropractic, the student-led IPE Council at the Canadian Memorial Chiropractic College has used the "Diamond Approach" to incorporate learning theories into voluntary IPE initiatives in partnership with medical students at the University of Toronto [14]. The "Diamond Approach" receives its name by virtue of design. It attempts to engage the 3 types of learners (auditory, visual and kinaesthetic) through a mixed module including an interactive exchange between medicine and chiropractic to support integrated health care practices. In addition, the school offers some exposure to interdisciplinary environments such as hospitals on a limited and voluntary basis.

## IPE Initiatives at McMaster University Medical School

In Canada, the Faculty of Health Sciences at McMaster University first incorporated programs in medicine, nursing, physiotherapy, occupational therapy, and midwifery in one integrated faculty.

Currently at the McMaster University medical school, students are required to fulfill IPE credits both during pre-clerkship and clerkship sections of their studies. IPE during pre-clerkship at the medical school takes the form of at least two half-day experiences where students shadow another health care professional. Students are able to choose from a variety of placements with physiotherapists, occupational therapists, registered dieticians, speech-language therapists, and social workers as well as any other local IPE preceptors who are interested in working with students.

While completing their clerkship, medical students have the opportunity to become involved in IPE activities and experiences throughout their clinical rotations as they are actively working in the health care environment and may encounter other health care professionals in the process of delivering patient care. For example, when completing patient rounds in a hospital, the student may be involved in discussions with members of a patient's health care team, which may be comprised of nurses, physicians, social workers, physiotherapists, occupational therapists or other health care professionals.

One example of a formalized IPE activity that occurs during clerkship is the opportunity for students to work with either nursing or midwifery students to complete simulated obstetrics and gynecology activities. This provides a platform to appreciate different scopes of practice and to generate discussions surrounding patient care issues or situations. In addition, as part of their family medicine clerkship rotation, students are required to complete a half-day interprofessional shadowing experience with health care providers such as chiropractors, osteopaths, naturopaths, and practitioners of traditional Chinese medicine.

Residency teaching units in the Department of Family Medicine at McMaster University have incorporated nurse practitioner-physician led teams since the early 1970 s. In 2006, two of the academic units have transitioned to a FHT. This transition enabled health professionals from other disciplines to join the existing interprofessional teams. The FHT has provided the opportunity to expand IPE programming for family medicine residents, undergraduate medical students and learners from other health professions [15].

## Argument for Interprofessional Shadowing to Enhance Team-Building Skills

At the Rosedale Medical Group site in Hamilton, Ontario both the community pharmacist and chiropractor have been co-located within the FHT environment for greater than five years and have hosted learners for over four years [16]. This opportunity for discussion and observation of the role of another health professional is an extension of IPE as well as a model for developing team-building skills for future clinical practice.

As part of their site visit evaluation a pharmacy student wrote the following regarding an interprofessional shadowing experience:

*"Having observed first-hand the seamless transition of care between pharmacists and chiropractors within the Family Health Team, I am truly inspired by the influence of a well-rounded professional team on patient health. I was delighted to learn that you work closely with the pharmacist to compare drug and non-drug approaches to pain management and am convinced that such discussion results in enhanced health outcomes for patients. In particular, it is this demonstration of proactive and consistent communication that facilitates an interprofessional approach to optimizing patient care on a daily basis*.

This experience has highlighted for me the way in which the chiropractic profession and that of pharmacy complement one another to facilitate comprehensive patient care. The referral of care between pharmacist and chiropractor is essential as effective pain management demands both physical manipulation to treat the underlying cause in conjunction with symptomatic drug treatment. I have learned that it is crucial to develop a strong support network amongst health care professionals at a time when collaborative care is so clearly recognized for its synergistic impact on patient health."

This instance offers an important practical argument for the use of interprofessional shadowing experiences between two professionals with seemingly separate roles. In this example, the pharmacy student spends a half-day observing the chiropractor that is treating patients. There is a brief introduction beforehand on the types of services chiropractors provide, some discussion on the efficacy of various interventions described in the literature, an explanation of relative and absolute contraindications to treatment, and a description of the encounter process for the half-day. While the chiropractor is rendering treatment the patient often engages in discussion about overlaps of care that the patient perceive are meaningful to the needs of the student. A brief summary discussion occurs among the chiropractor and pharmacy student between patients about the cases they just observed. On completion of the half-day, the pharmacy student poses any remaining questions to the chiropractor and submits a written self-reflection summary of their experience to the chiropractor and to the affiliated coordinator at the educational institution.

Patient-centered approaches, shared medical records and provider co-location are important facilitators of collaborative care that help equip multi-disciplinary clinical settings to host learners. The IPE initiatives at the residency teaching units in the Department of Family Medicine at McMaster University also identify the following collaboration enablers: a common team room to facilitate interaction amongst providers and learners; information technology to facilitate rapid communication and care planning; strong and consistent leadership; flexibility of administrative operations; and cultural support [15]. Lastly, respect and trust amongst health care professionals is an essential precursor to ensuring effective collaborative care in these environments [17].

## Learner-Derived and Active Shadowing Experiences

For interprofessional shadowing experiences it is recommended that the educational institution not define the specific site visit objectives. An adult learning approach is favoured. However, the main learning outcomes for this intervention could be explicitly described by the institution to ensure learner participation in these goals, such as identifying profession-specific terminology, benefits and challenges of team-building and interprofessional communication. The opportunities for the student to gain insight into different approaches to patient assessment, profession-specific terminology and to establish their own team-building expertise are components of effective IPE [18].

This approach demonstrates the value of letting the learner take an active role in defining the objectives specific to his or her needs. Students can be encouraged to explore ways in which health professionals may effectively communicate with one another in order to further improve patient care. Understanding each profession's scope of practice helps to ensure that the patient sees the right team member at the right time. This facilitates timely, clear and consistent communication.

For example, a possible method to further evolve adult learning into a more active approach for clinical shadowing involves six steps:

1. Summarize history and findings

2. Narrow the differential

3. Analyze the differential

4. Probe preceptor about uncertainties

5. Plan management

6. Select case-related issues for self-study

The SNAPPS model [19], which was first implemented and studied at Case Western Reserve University School of Medicine, is intended to allow the student to become an active learner in their clinical encounters with patients by encouraging the student to take a lead role in their learning. The student is able to utilize their preceptor as a content expert and facilitator to assist them in developing personal learning objectives. A randomized comparison group trial of students using the SNAPPS model found that it facilitates enhanced expression of diagnostic reasoning during case presentations [20].

A modified SNAPPS model could be used during an IPE placement with students whose health care education is a component of a second educational degree. This approach matches recent IPE systematic review findings that favor adult learning approaches [21]. This approach would allow the student to focus on cases of interest, either by interviewing the patients alone or under their preceptor's supervision. When presenting these cases to their preceptor, the student would then be able to discuss their diagnostic reasoning and management plan. This active learning in turn helps the student to develop further learning possibilities. In interprofessional shadowing experiences where professionals have little knowledge of each other's field of expertise, SNAPPS can also assist in bridging differences in learning objectives between diverse professions who have minimal overlaps in scopes of practice. For example, a pharmacy student, in the context of shadowing a chiropractor, can gain insight into the non-drug musculoskeletal approaches to patient therapy.

A pharmacy student shadowing a chiropractor will be able to take an active role in the approaches to managing musculoskeletal conditions from a non-pharmacological perspective. Furthermore, the pharmacy student can broaden their awareness of non-pharmacological approaches to pain management in cases where drugs may be contraindicated. Some examples include:

• during pregnancy

• when adverse drug reactions have occurred

• being at maximum doses of pain medication

• for patients who prefer a non-pharmacological approach

The student would be able to develop team-building skills around which types of patients or patient problems can benefit from chiropractic care and how recommending such musculoskeletal treatments may be incorporated into the Pharmaceutical Care Process [22] as an adjunct or alternative for pain management in the care of future patients. This interprofessional shadowing experience emphasizes the clinical utility of team-building in developing a strong support network amongst health professionals to which one's own patients can be referred when interventions become limited.

In the overall development of interprofessional shadowing experiences, three key factors have been found to influence collaborative practice: interaction factors; organizational factors; and systemic factors [23].

There is a paucity of chiropractic literature on IPE. Fortunately, it is not necessary to "re-invent the wheel" if an institution is new to IPE. Curriculum and program modules from other health professional programs that are more established in IPE can be used as a strong starting point. Authors from other professions have previously noted that the planning and implementation of an interprofessional shadowing placement should account for both internal (group-based) and external (management and organizational) issues at the outset [24]. Lastly, measures of effectiveness over time could also be considered [25].

## Conclusion

Interprofessional primary care settings are uniquely positioned to provide IPE. The formal interprofessional shadowing instance described between a pharmacy student and chiropractor offers some insight for the value of including additional, typically, less diverse professions into collaborative care models. In recognition of the strategic importance of health workforce resources and expanding interprofessional models of care, private institutions, that only offer chiropractic education, should implement mandatory interprofessional shadowing experiences in multi-disciplinary clinical settings.

One assumption made was that the local jurisdiction described is reflective of IPE initiatives by the chiropractic profession in other geographical areas. This assumption was rendered to emphasize an apparent dearth of chiropractic literature in this area. There may indeed be instances of IPE initiatives occurring at chiropractic institutions that have not been documented in the literature. A global scoping review of chiropractic IPE initiatives would be a reasonable next step.

## List of abbreviations

IPE: Interprofessional Education; FHT: Family Health Team; SNAPPS: Summarize history and findings; Narrow the differential; Analyze the differential; Probe preceptor about uncertainties; Plan management; Select case-related issues for self-study

## Competing interests

The authors declare no competing interests. No funds were received for the preparation of this manuscript. No author has any financial interest in or benefit from this research.

## Authors' contributions

JR, JL and ES initiated the project. JR provided the chiropractic IPE initiatives concept. JL and IK provided the pharmacy IPE initiates concept. ES and AM provided the medical school IPE initiatives concept. AE and JL provided team-building concept and student quotation. ES, JL and AM provided learner-derived objectives concept. All authors reviewed entire manuscript for edits, feedback and approval.

## Authors' information

JR is a practicing chiropractor and faculty member at McMaster University medical school. JL is a pharmacy resident from the school of Pharmacy at the University of Toronto. ES is a medical student at McMaster University medical school. AM is a practicing family physician and faculty member at McMaster University medical school. AE is the health policy analyst for the Ontario Chiropractic Association. IK is a community pharmacist and Chair of the Board of Directors for Dell Pharmacy.

## Author details

^1^Department of Family Medicine, McMaster University, McMaster Innovation Park, 175 Longwood Rd S, Hamilton, ON, Canada. ^2^Toronto Western Hospital, 399 Bathhurst St, Toronto, ON, Canada. ^3^Michael G. DeGroote School of Medicine, McMaster University, Niagara Regional Campus, 142 Queenston St, St Catharines, ON, Canada. ^4^Ontario Chiropractic Association, 20 Victoria St, Toronto, ON, Canada. ^5^Dell Pharmacy, Rosedale Medical Group, 1955 King St E, Hamilton, ON, Canada.
